# The temporal frequency tuning of continuous flash suppression reveals peak suppression at very low frequencies

**DOI:** 10.1038/srep35723

**Published:** 2016-10-21

**Authors:** Shui’er Han, Claudia Lunghi, David Alais

**Affiliations:** 1 School of Psychology, University of Sydney, NSW 2006, Australia; 2 Department of Translational Research on New Technologies in Medicine and Surgery, Via Savi 10, 56100 Pisa, Italy; 3 Neuroscience Institute, National Research Council (CNR), Via Moruzzi 1, 56100, Pisa, Italy

## Abstract

Continuous flash suppression (CFS) is a psychophysical technique where a rapidly changing Mondrian pattern viewed by one eye suppresses the target in the other eye for several seconds. Despite the widespread use of CFS to study unconscious visual processes, the temporal tuning of CFS suppression is currently unknown. In the present study we used spatiotemporally filtered dynamic noise as masking stimuli to probe the temporal characteristics of CFS. Surprisingly, we find that suppression in CFS peaks very prominently at approximately 1 Hz, well below the rates typically used in CFS studies (10 Hz or more). As well as a strong bias to low temporal frequencies, CFS suppression is greater for high spatial frequencies and increases with increasing masker contrast, indicating involvement of parvocellular/ventral mechanisms in the suppression process. These results are reminiscent of binocular rivalry, and unifies two phenomenon previously thought to require different explanations.

The recent couple of decades has seen a great deal of research activity aimed at elucidating the extent to which visual processing can occur outside of conscious awareness. Several approaches have been used including binocular rivalry^1^,^2^ and various kinds of masking^3^. More recently a new approach known as continuous flash suppression (CFS) has become very popular since it first appeared^4^,^5^,^6^. Like binocular rivalry, it involves presentation of irreconcilable images to each eye which prevents binocular fusion and triggers suppression of one eye’s image. In the case of CFS, one eye receives a sequence of dynamic ‘Mondrian’ images updated typically at a rate of 10 Hz which reliably suppresses a target image of low to moderate contrast in the other eye (Fig. 1a). The main appeal of CFS is that it provides a very strong and long-lasting suppression and the initial percept is reliably the dynamic masker. In contrast, binocular rivalry typically involves two dichoptic static images, which perceptually alternate in a stochastic manner. This allows easy study of visual processing in the suppressed eye and has seen CFS rapidly become the standard tool for investigating visual processing outside of awareness^5^,^7^,^8^.

The mechanisms underlying CFS are not well understood and it is not clear why the dynamic Mondrian pattern provides such strong masking. This has not stopped many CFS studies from publishing bold claims about what kinds of information are processed in the absence of conscious awareness, including: preferential access to awareness for alphabets from native languages, upright, fearful and familiar facial stimuli, and reduced aftereffects specific to early stimulus properties^4^,^8^,^9^,^10^,^11^. Nevertheless, these findings have not been found to be robust^12^,^13^,^14^ and differ from those obtained using binocular rivalry which generally show that images presented to the suppressed eye undergo very little processing^15^,^16^,^17^,^18^. This difference is curious given that CFS is often presumed to be a form of interocular suppression, like binocular rivalry.

A better understanding of the mechanisms of CFS suppression is needed to clarify the role of interocular suppression and possible links between CFS and binocular rivalry. This is all the more important because of the strong theoretical implications of claims about images that are imperceptible nonetheless undergoing visual processing and reaching awareness. A clear understanding of the suppressive mechanisms involved and appropriate stimulus control are critically important in validly drawing such conclusions. As a starting point, we investigate the temporal frequency tuning of CFS using temporally narrow-band maskers, a dimension that has not been systematically studied previously. Although previous studies have examined the effect of varying Mondrian refresh rates^4^,^19^, frequency analyses show that the spectrum is consistently broadband and low-pass (Fig. 1c,d). Consistent with these observations, we find that masker suppression–when tested with narrowband temporal modulations–peaks very prominently at approximately 1 Hz, well below the 10–15 Hz refresh rates typically used in CFS studies^20^,^21^,^22^.

## Results

### Experiment 1

We examined the effect of temporal frequency in narrowband maskers (1 octave full-width at half-height; see also Fig. 2b) on CFS suppression duration. Temporal frequencies were centred at rates of 0, 0.375, 0.75, 1.5, 3, 6.25, 12.5 and 25 Hz. Data were analysed in a one-way (masker temporal frequency) repeated-measures ANOVA. The effect of masker frequency was highly significant, with *F*(6, 72) = 17.5, *p* < 0.0001, η_p_^2^ = 0.59, and results are plotted in Fig. 3a as normalized suppression duration in a semilog plot. Consistent with the frequency spectrum of the Mondrian, suppression was much stronger at low than at high frequencies, yet the pattern follows a bandpass tuning that was well described by a Gaussian normal function. The Gaussian was fitted using a maximum likelihood routine with three free parameters: mean, standard deviation and vertical offset. Amplitude was not a free parameter and was defined as the maximum normalised suppression duration minus the baseline. The best-fitting Gaussians had the following parameters: mean = 0.97 Hz (SD = 0.48), standard deviation = 1.42 octaves (SD = 0.63), baseline = 0.44 (SD = 0.20) and amplitude = 1.52 (SD = 0.59).

To contrast the effect of masking temporal frequency with the static masker we binned the temporal frequency data into two categories of low and high frequency maskers. From Fig. 3a, it is clear that 0.375, 0.75, 1.5 and 3 Hz fall within the same passband whereas the remaining frequencies, 6.25, 12.5 & 25 Hz, do not. This accords with previous research that estimated a broad, lowpass channel below 5 Hz and bandpass channel (>6 to 20 Hz) in human vision^23^,^24^. We therefore binned 0.375, 0.75, 1.5 and 3 Hz together as ‘low frequency’ and the remaining masker frequencies were defined as ‘high frequency’. Figure 3b shows the mean normalised suppression durations for the low and high masker temporal frequencies and the static control. A two-tailed paired *t*-test revealed that slow modulating maskers produced significantly larger normalised suppression durations than the static control, *t*(12) = 3.38, *p* < 0.01, confirming what is clear from the tuning function plotted in Fig. 3a. Interestingly, fast modulating maskers were less effective than a static mask, producing shorter suppression durations than the static control, *t*(12) = 3.53, *p* < 0.01.

To validate the relevance of these trends to the CFS literature, we compared the peak raw suppression durations with that of a standard 10 Hz Mondrian masker^25^. To obtain the peak suppression duration of each participant, a Gaussian normal function was first fitted to the raw data with amplitude as an additional free parameter. Peak suppression duration was then computed by summating the fitted amplitude and baseline. As depicted in Fig. 3c, two tailed independent t-tests revealed that the most effective low frequency masker was comparable to the 10 Hz Mondrian masker, *t*(23) = −0.28, *p* = 0.78, *d* = 0.12. To further assess the performance of the filtered noise maskers, we computed the within-subject variability in raw suppression durations for low and high frequency bins and compared the results with that of the 10 Hz Mondrian. Two tailed independent t-tests revealed less variable raw durations for the low and high frequency noise maskers, *t*(23) = 2.26, *p* < 0.05, *d* = 0.94 and *t*(23) = 4.35, *p* < 0.001, *d* = 1.81 respectively, demonstrating a methodological advantage with filtered noise maskers.

### Experiment 2

We measured the temporal contrast sensitivity function for our temporally filtered masking stimuli to determine whether the peak suppression at low temporal frequencies was simply a manifestation of the tuning towards low temporal frequencies in human vision. Human contrast sensitivity to temporal frequency shows a lowpass bias^26^ and is in general more sensitive to contrast changes at lower temporal frequencies with a corner frequency at around 8–10 Hz^27^,^28^. As these observations were made with narrow-band (sinusoidal) modulations of sinusoidal gratings, we wanted to establish the temporal contrast sensitivity function for our masker stimuli, which differ considerably to these classical stimuli in being spatially broadband noise patterns with an inverse frequency spectrum and having a temporal modulation bandwidth of one octave.

Absolute and increment contrast thresholds were measured for our masker stimuli and performance was compared using a Wilcoxon Signed-Ranks test. This revealed the resulting temporal contrast sensitivities for the two measures did not differ significantly (*Z* = 1.26, *p* = 0.21) and thus the sensitivity curves were combined and averaged for each participant. The group-averaged temporal sensitivity function is plotted in Fig. 4. A one-way, repeated-measures ANOVA and revealed a significant effect of temporal frequency, *F*(6, 54) = 37.4, *p* < 0.0001, η_p_^2^ = 0.81. The data were further explored using trend analyses up to third order and were found to exhibit a strong quadratic and a significant cubic trend, *F*(1, 9) = 228.6, *p* < 0.0001, η_p_^2^ = 0.96, *F*(1, 9) = 45.7, *p* < 0.001, η_p_^2^ = 0.84, respectively, but no significant linear trend, *p* > 0.05.

To compare the temporal contrast sensitivity function with the temporal frequency suppression tuning observed in Experiment 1, we conducted a two-way ANOVA with the factors being temporal frequency and task type (normalised suppression durations vs. normalised contrast sensitivity values). The main effect of temporal frequency was significant, *F*(6, 126) = 25.2, *p* < 0.0001, η_p_^2^ = 0.55 as was the main effect of task type, *F*(1, 21) = 146, *p* < 0.0001, η_p_^2^ = 0.87. The important result was that temporal frequency interacted significantly with task, *F*(6, 126) = 21, *p* < 0.001, η_p_^2^ = 0.5, showing that temporal contrast sensitivity does not explain the temporal tuning in Experiment 1. As indicated in Fig. 4, pairwise contrasts (Holm-Bonferroni corrected) showed the significant interaction was due to lower CFS suppression durations at 6.25 and 12.5 Hz, 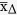 = −1.66, and −1.07, *t *=* −*10.3 and −11 respectively, *p* < 0.001, and higher suppression durations at 0.375 and.75 Hz, 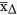 = 0.22, *t* = 1.55, *p* < .01 and 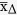 = 0.28, *t* = 1.47, *p* < 0.05 respectively.

### Experiment 3

The confirmation that CFS suppression peaks at a very low temporal frequency is very informative as it suggests the involvement of the parvocellular pathway^29^,^30^ in CFS suppression. Neurons in this pathway are most sensitive to low temporal frequencies^31^,^32^,^33^ and high spatial frequencies, peaking around 6–10 cpd^27^,^31^. Interocular suppression in binocular rivalry is also thought to have a parvocellular basis^32^,^34^,^35^. Experiment 3 tested whether CFS suppression exhibits a bias for high spatial frequencies by comparing suppression durations for targets of low (1 cpd) and high (10 cpd) spatial frequency using the same masking stimuli as in Experiment 1. As these maskers have a 1/f spatial frequency profile, they produce equivalent neural response to all spatial frequencies which ensures the two spatial frequency conditions are comparable.

The results of Experiment 3 are plotted in Fig. 5 and were analysed in a within-subjects, two-way (masker temporal frequency x spatial frequency) repeated-measures ANOVA. There were significant main effects for masker temporal frequency, *F*(6, 66) = 14.2, *p* < 0.0001, η_p_^2^ = 0.56, and target spatial frequency, *F*(1, 66) = 39.3, *p* < 0.0001, η_p_^2^ = 0.78. As expected, there was a significant interaction between masker temporal frequency and target spatial frequency, *F*(6, 66) = 8.4, *p* < 0.01, η_p_^2^ = 0.43. Pairwise comparisons with Holm-Bonferroni corrections demonstrated greater suppression durations for high spatial frequency targets at every level of temporal frequency (see Table 1 for statistical details).

As in Experiment 1, Gaussian distributions were fitted to the group mean data (Fig. 5a) and to individual subjects’ data. Two participants’ data were excluded because all suppression times were uniform for the low spatial frequency condition and the Gaussian could not be meaningfully fitted. For the 10 remaining participants the parameters for the best-fitting individual Gaussians were compared across the two levels of spatial frequency using Holm-Bonferroni corrected paired t-tests (see Table 2 for details). No significant differences were found for the Gaussian mean, standard deviation or baseline elevation, but a large and significant difference was obtained for amplitude, with high spatial frequency targets exhibiting longer suppression durations, *t*(9) = 4.57, *p* < 0.01, consistent with high spatial frequencies selectively activating CFS suppression mechanisms.

To contrast temporally modulating and static maskers, we binned temporal frequency into low and high frequency groups (as in Experiment 1) and compared them against the static masker, for both levels of spatial frequency (Fig. 5b). The data for high spatial frequencies replicated the pattern observed in Experiment 1 (Fig. 3b): slow modulating maskers produced significantly higher suppression durations than a static masker, *t*(11) = 2.88, *p* < 0.05 and fast modulating maskers produced significantly lower suppression durations, *t*(11) = 3.98, *p* < 0.01. For low spatial frequency targets, suppression duration was significantly lower with fast modulating maskers compared to static maskers, *t*(11) = 4.13, *p* < 0.01, but there was no significant difference between static and slow modulating maskers, *t*(11) = 0.03, *p* = 0.98. These comparisons are summarised in Fig. 5b.

### Experiment 4

As well as being tuned to low temporal and high spatial frequencies, another characteristic of parvocellular processes is their contrast sensitivity. While the magnocellular contrast response function rises steeply but saturates early at around 20–30% contrast, the parvocellular contrast response function exhibits a steady and non-saturating increase^36^,^37^. Experiment 4 tested the effect of masker contrast at three levels (30, 50 & 90%) on suppression duration, for masker modulation rates of 2 and 10 Hz. If the low temporal frequency bias in suppression durations seen in Experiment 1 is due to the involvement of parvocellular mechanisms in CFS suppression, a slow modulating masker should produce gradually increasing suppression durations as masker contrast increases, with no saturating plateau. A two-way (masker temporal frequency x masker contrast), repeated-measures ANOVA was run on the normalized data. Masker temporal frequency and masker contrast interacted significantly, *F*(2,22) = 7.06, *p* < 0.05 η_p_^2^ = 0.39. The interaction was due to significantly greater suppression durations for the 2 Hz masker compared to 10 Hz across different contrast levels (Fig. 6b). The differences between masker rates were compared with Holm-Bonferroni corrected t-tests and were significant across all contrast levels, *t*(11) = 7.05, *p* < 0.001, *t*(11) = 3.30, *p* < 0.01, *t*(11) = 3.71, *p* < 0.01, in ascending order of contrast. Separate analyses for each masker frequency showed a significant main effect of masker contrast for the 2 Hz masker, *F*(2,22) = 10.85, *p* < 0.001, η_p_^2^ = 0.50, but not for the 10 Hz masker, *F*(2,22) = 0.81, *p* = 0.41, η_p_^2^ = 0.07. For the slow modulating masker, suppression durations followed a significant increasing linear trend, *F*(1,11) = 15.7, *p* < 0.01, η_p_^2^ = 0.59.

## Discussion

CFS is widely used to study unconscious processing of visual images suppressed from awareness yet little is known about the suppression process and appropriate stimulus control is lacking. We used spatiotemporally filtered dynamic noise sequences to reveal the temporal frequency tuning of CFS. Surprisingly, given the widespread use of Mondrian flicker rates in the range of 10–15 Hz, the temporal tuning peaks at about 1 Hz and is clearly bandpass, with suppression declining on either side of the peak–particularly on upper side. Indeed, maskers modulating at a typical CFS rate of 12.5 Hz provided very weak suppression and were less effective than a static noise image. Complementing the observed low-temporal-frequency bias, CFS suppression was stronger for high-spatial-frequency targets and increased monotonically with masker contrast.

**Figure 1:**
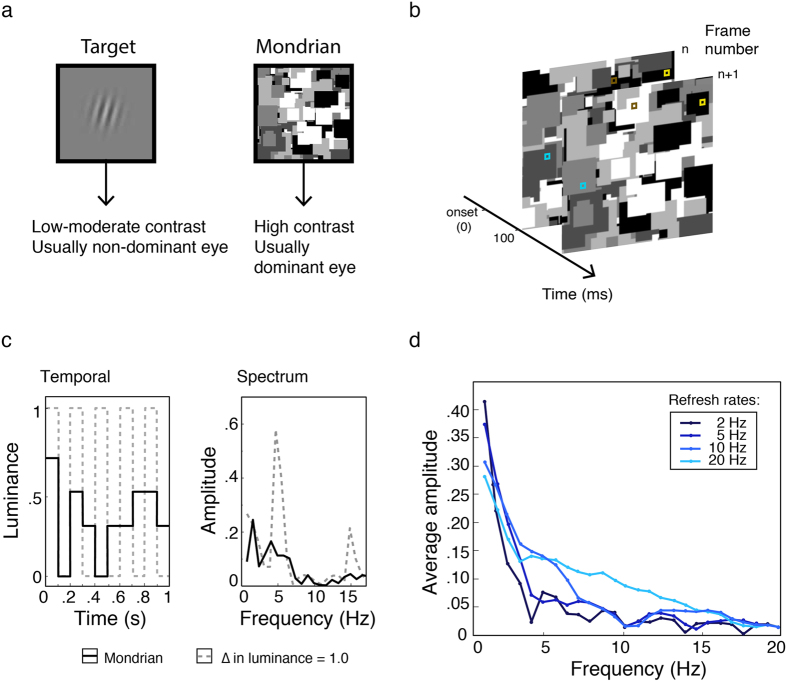
(**a**) Traditional CFS involves a dynamic Mondrian composed of randomly positioned shapes varying in size and luminance presented usually to the dominant eye and a smaller target to the other. Targets may be natural images or simple stimuli. (**b**) Dynamic Mondrians are commonly updated at 10 Hz by presenting new patterns every 100 ms. Because the grey levels of the Mondrian shapes vary randomly, some undergo large luminance changes between patterns (brown squares) whereas others change little or not at all (blue and yellow squares, respectively). Statistically, over a sequence of frames, the latter is much more likely, and this lengthens the period of the modulation and thus lowers the frequency. (**c**) Even if strong alternations did occur between the extreme luminance values, a 10 Hz Mondrian update rate would produce a 5 Hz square-wave modulation (grey dashed line, left) with a peak at 5 Hz and lesser peaks at the odd harmonics (grey dashed line, right). The actual waveform, however, is inevi complex with low frequency components due the presence of multiple grey levels (here, *n* = 5) and non-uniform changes over time (black solid line, left). Consequently, the temporal spectrum is broader with a concentration of energy at frequencies much lower than the intended update rate (black solid line, right). (**d**) To demonstrate the low-frequency bias, we tracked the pixel timelines of 70 grayscale Mondrian patterns updated at 2, 5, 10 and 20 Hz (randomly sampling from 5 grey levels, 200 pixels each refresh rate), then Fourier transformed the data. The resultant amplitude spectra for all refresh rates have a very strong low-pass profile. For the typical 10 Hz Mondrian, only 1.3% of total stimulus energy occurs at 10 Hz and the peak frequency occurs at 1 Hz, which has more than 20 times the energy (31%) of the 10 Hz component. Raising the Mondrian update rate does little to boost high-frequency content and the strongly lowpass profile remains. Indeed, as the functions decline with frequency, they could be well described as temporal “pink noise”.

The key to our stimulus is that it is narrowband. Even though a modulation of 1 Hz is quite slow, our stimulus modulated smoothly to provide uniform masking over time, in contrast to the discrete modulation of a dynamic Mondrian which is intermittently updated. Some studies have compared various Mondrian update rates^4^,^19^ but this is not equivalent to manipulating temporal frequency in a pattern that varies randomly in luminance between updates. The reason is simply that the typical presentation time of each Mondrian pattern i.e., 100 ms lengthens the period of modulation, producing a slower alternating waveform than intended. Moreover, given that the probability of maximal luminance alternations between successive updates is very low (i.e., *n*^*−*10^ for ten successive patterns with *n* fixed grey levels), power inevitably concentrates at lower temporal frequencies. This is illustrated in Fig. 1c,d, where several very different flicker rates produce very similar frequency profiles, all concentrated at very low frequencies, with only a small fraction of the temporal energy present at the nominal flicker frequency. Even in the unlikely case that the stimulus alternated between the extreme luminance values from frame to frame, for example at a typical Mondrian rate of 10 Hz, this would create a square-wave modulation with a peak frequency at half the nominal flicker rate, that is, at 5 Hz. Here, by using spatiotemporally filtered noise as maskers we gain full and independent control over the spatial and temporal dimensions, without significant loss of suppressive strength despite the loss of contours and edges^38^,^39^ (Fig. 3c). This accords with evidence showing effective suppression in CFS by low-pass filtered Mondrians^5^, broad tuning functions for Mondrian refresh rates^19^ and robust suppression in binocular rivalry with filtered noise^40^,^41^. Moreover, our results advance previous work with broadband stimuli^5^,^38^ in characterising the nature of the mechanisms underlying CFS.

The stimulus tunings we report for CFS are consistent with a suppression process dominated by input from the parvocellular/ventral pathway. The parvocellular and the magnocelluar pathways are the two major paths from retina to cortex. They are well segregated up to V1 and after some interaction in early cortex, these subcortical paths project with a bias to the two major cortical pathways: parvo to the ventral stream and magno to the dorsal stream^42^. Consistent with the selectivity we report for CFS suppression, parvo/ventral neurons are tuned to low temporal frequency, high spatial frequency^27^,^31^ and the contrast response function has a steady and non-saturating increase with contrast^36^,^37^. By contrast, magno/dorsal neurons are tuned to high temporal and low spatial frequency stimuli, rising steeply with contrast but saturating early at around 20–30% contrast^29^,^30^. The finding that CFS suppression has a bandpass temporal tuning centred at a very low frequency, tuned to high spatial frequency and gains steadily in strength with increasing contrast suggests the involvement of the parvocellular pathway.

Our findings help clarify the CFS literature, which has been overly concerned with the rapid flicker in CFS maskers when seeking to explain CFS suppression. CFS suppression with flickering Mondrians has been described as driven by a continued barrage of transients^7^, which may also reduce the retinotopic neural adaptation in the masking eye^4^,^5^. According to this interpretation, higher Mondrian refresh rates are therefore required to generate more transients and to reduce retinotopic adaptation. While flicker will certainly generate transients, and might attenuate retinotopic adaptation, this is clearly secondary. By far the biggest component of CFS suppression comes from low temporal frequencies, which are overwhelmingly the largest components in the temporal frequency spectrum of a flickering Mondrian (Fig. 1d). This analysis alone suggests that CFS suppression is likely driven most strongly by maskers modulating at low temporal frequency, a observation that tallies with evidence showing the dominance of natural stimulus properties in rivalry^39^,^43^. Our results confirm this, and together with the spatial frequency and contrast findings, suggest that the parvocellular/ventral pathway will be critical for an explanation of CFS suppression.

The likely involvement of parvocellular/ventral mechanisms in CFS links suppression in CFS more closely with binocular rivalry suppression–which is also thought to have a parvocellular/ventral bias^24^,^25^,^26^,^27^,^28^,^29^,^30^,^31^,^32^,^33^,^34^. Binocular rivalry studies have shown that motion information can be integrated across two eyes even while they engage in colour and form rivalry^44^,^45^,^46^,^47^, suggesting no suppression of conflicting monocular motion signals. Thus both CFS and binocular rivalry suppression likely involve parvocellular/ventral mechanisms. This is parsimonious at a theoretical level as both involve interocular suppression and unifies two phenomenon previously thought to require different explanations^7^. Moreover, it provides a possible framework for studying CFS, a proposition that is also supported by similar contributing factors (e.g., feature selectivity)^5^,^38^,^47^ to both types of suppression. The one obvious difference is that CFS and binocular rivalry produce vastly different perceptual experiences, as unlike binocular rivalry, perception in CFS does not alternate between the competing images^4^. A straightforward explanation of this could simply be that CFS involves a great imbalance of stimulus strength between the two eyes and perception follows the strongest image. This is true in binocular rivalry, where a contrast imbalance between the images will bias dominance to the stronger image and, notably, CFS studies invariably use a high-contrast masker and a target of low-to-moderate contrast. Consistent with this idea, we showed in Experiment 4 that raising CFS masker contrast did prolong masker dominance (Fig. 4).

The current study mapped the temporal tuning of CFS suppression with temporally narrowband maskers. Our results revealed a low, bandpass temporal frequency tuning function that becomes more pronounced for high target spatial frequencies and increasing masker contrast. Similar to binocular rivalry^32^,^33^,^34^,^35^, these results indicate a parvocellular/ventral pathway involvement in CFS, opening up explanatory accounts of CFS to the more widely modelled phenomenon of binocular rivalry^48^,^49^,^50^,^51^,^52^,^53^. Our results also show that CFS is not simply a convenient method for suppressing visual awareness. Instead, it is a paradigm highly sensitive to the spatiotemporal properties of a stimulus and inappropriate stimulus control could weaken suppression, increase the impact of response biases and demand characteristics and complicates the type of conclusions that can be drawn from it^54^,^55^,^56^. The use of spatiotemporally filtered noise is one way to provide proper stimulus control, and thus possesses a methodological advantage in uncovering the characteristics of CFS suppression. Future psychophysical and imaging studies using spatiotemporally filtered dynamic noise will further elucidate the neuronal processes underlying CFS.

## Methods

### Participants

All participants were drawn from a pool of undergraduate students at the University of Sydney studying second or third year psychology courses and were reimbursed $AU20 per hour for their participation. All had normal or corrected-to-normal eyesight. Participants also had normal stereovision, assessed using the Fly Stereo Acuity test. Experiments accorded with the Declaration of Helsinki and were approved by the University’s Human Research Ethics Committee. Informed consent was also obtained from all participants. Samples were as follows:- Experiment 1: Author SH plus twelve naive observers (age range: 18–30 years, *SD *=* *4.19 years, 10 females) completed the task with filtered noise maskers whereas twelve naive observers (range: 19–30 years of age, *SD *=* *4.33 years of age, 9 females) were presented with the Mondrian; Experiment 2: Author SH plus nine naive observers (range: 19–29 years of age, *SD *=* *4.28 years of age, 8 females); Experiment 3: Twelve naive observers (range: 19–30 years of age, *SD *=* *3.75 years of age, 8 females); Experiment 4: Twelve naive observers (range: 19–30 years of age, *SD *=* *4.33 years of age, 9 females).

## Experiment 1

### Masker stimuli

**Figure 2:**
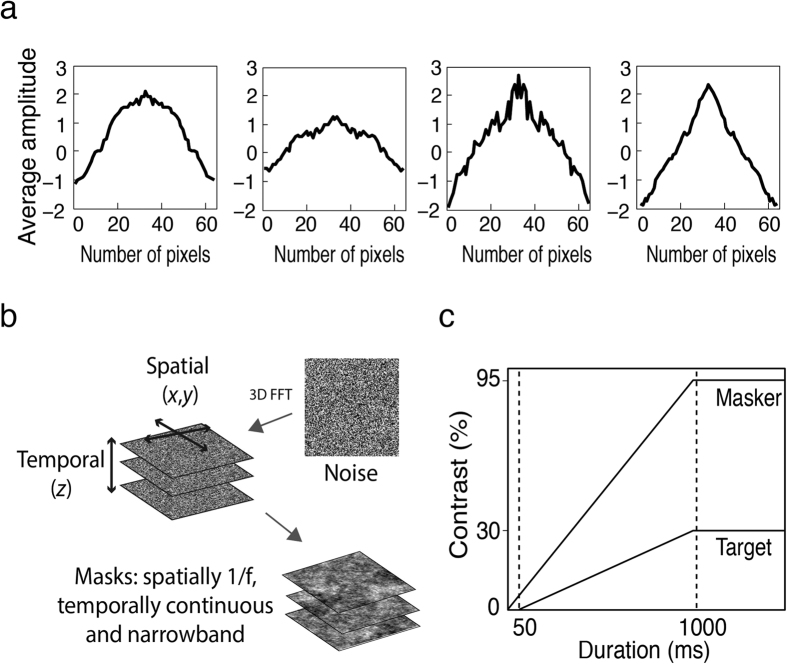
(**a**) Amplitude spectrum of target images: For comparability with previous studies, we used four greyscale natural images in Experiment 1. To select an appropriate masker, these images were first analysed with a two-dimensional FFT. The results reveal a 1/f spatial profile for each of the target images, thus maskers were given a 1/f amplitude spectrum. (**b**) Spatiotemporal filtering: A three-dimensional FFT was first computed for a stack of 205 randomly generated noise images. Filtering the z-axis with a log-Gaussian filter controlled masker temporal frequency, whereas individual noise images (x and y axes) were convolved with a circularly symmetric inverse frequency filter for spatial frequency. The resultant stimulus was temporally narrowband, continuously modulating pink noise. (**c**) Ramped onsets: To avoid abrupt transients, both masker and targets were ramped up to their maximum contrast over a period of 1000 ms and the masker preceded the target by 50 ms to allow cumulative suppressive effects.

**Figure 3:**
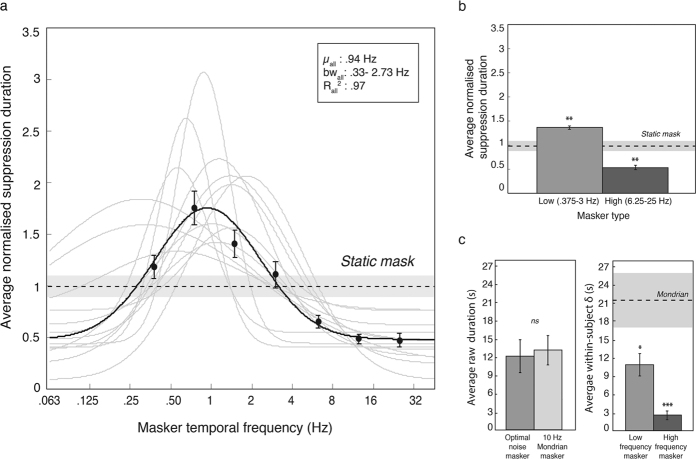
(**a**) Data from Experiment 1 showing suppression duration as a function of masker temporal frequency, with frequency plotted on a base 2 logarithmic scale. Maskers were dynamic noise stimuli filtered in the time dimension into narrow temporal frequency passbands. The data show CFS suppression duration is strongly dependent on temporal frequency, with maximum suppression for low temporal frequency maskers. The data pattern is consistent across all observers (grey traces) and is distinctly bandpass, not lowpass. With temporal frequency plotted on an octave scale (i.e., base 2 logarithm), the pattern is very well described by a Gaussian normal function. The best fit to the group data (solid black line: R^2^ = 0.97) has a peak at 0.94 Hz and a standard deviation of 1.42 octaves. Peak suppression frequency for individual participants ranged from 0.30–1.85 Hz. (**b**) The data binned into low (0.375, 0.75, 1.5, 3 Hz) and high (6.25, 12.5 25 Hz) temporal frequencies, contrasted against a static noise masker (dashed line with shaded error bar) shows faster maskers resulted in significantly lower suppression durations than the static condition. (**c**) Raw data analyses comparing group averages and standard deviations for filtered noise maskers with a standard 10 Hz Mondrian. Peak suppression durations estimated from fitted Gaussian functions (‘optimal noise masker’) were comparable to the Mondrian, with the added advantage of lower within-subject variability, as demonstrated by the significantly lower variance in low and high frequency maskers compared to the Mondrian. These results demonstrate the applicability of narrowband filtered noise maskers for studying CFS temporal frequency processes. Asterisks denote statistical significance (*p < 0.05, **p < 0.01, ***p < 0.001) after Holm-Bonferonni correction for multiple comparisons. Error bars represent ±1 standard error of the mean.

**Figure 4:**
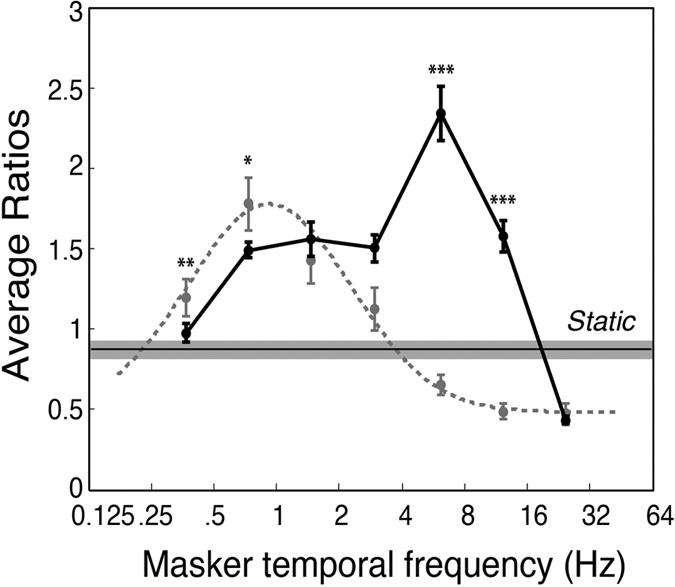
Data from Experiment 2. The solid black line shows temporal frequency sensitivity as measured in Experiment 2 to modulating noise patterns with a 1/f spatial frequency spectrum. For comparison, the suppression duration data from Experiment 1 together with the best-fitting function are replotted (dashed line). Temporal frequency is plotted on a base 2 logarithmic scale. The two datasets exhibit very different patterns and indicate that temporal contrast sensitivity cannot explain the temporal tuning of CFS suppression. Asterisks denote statistically significant differences between contrast sensitivity and suppression duration trends using independent samples t-tests, after Holm-Bonferonni correction (*p < 0.05, **p < 0.01, ***p < 0.001). Error bars show ±1 standard errors of the mean.

**Figure 5:**
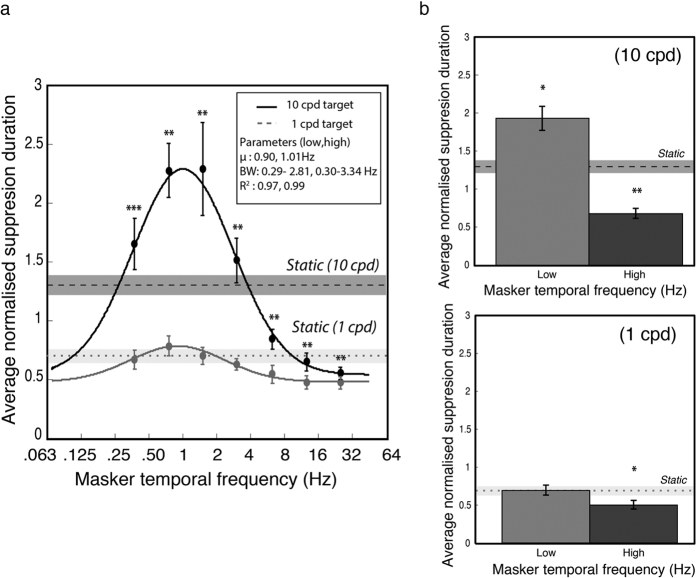
Data from Experiment 3. (**a**) Suppression duration as a function of masker temporal frequency and target spatial frequency. The suppressed target was either high spatial frequency (10 cpd, black line) or low spatial frequency (1 cpd, grey dashed line). Targets of 10 cpd were very strongly suppressed and showed a clear bandpass relationship over masker temporal frequency. Targets of 1 cpd showed significantly less suppression and were almost uniform over temporal frequency. The best-fitting Gaussians had similar peak frequencies and standard deviations for both low and high spatial frequencies: mean = 0.77 and 1.08 Hz, and standard deviation = 1.16 and 1.17 octaves, respectively. (**b**) Results for 10 and 1 cpd targets (upper and lower plots respectively), with low and high temporal frequencies contrasted against a static noise masker. Similar to Experiment 1, low frequency maskers suppressed 10 cpd targets for longer periods, and high frequency maskers for shorter periods, than the static condition. In contrast, low frequency maskers did not effectively suppress low spatial frequencies relative to a static masker, although faster maskers were found again to produce shorter suppression durations than the static condition. Asterisks denote statistical significance (*p < 0.05, **p < 0.01, ***p < 0.001) after Holm-Bonferroni correction. Error bars represent ±1 standard errors of the mean.

**Figure 6:**
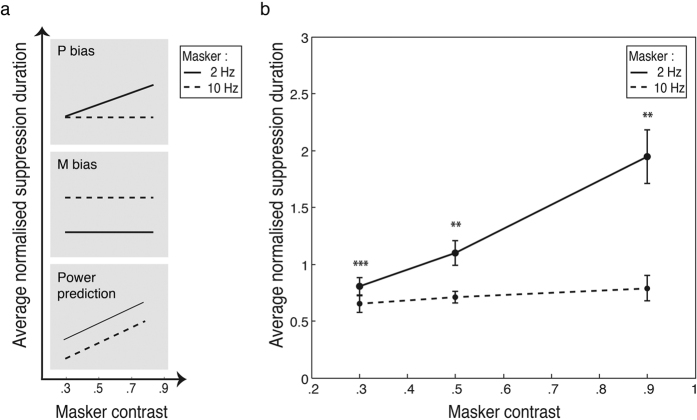
Predictions of contrast effect and results from Experiment 4. (**a**) If CFS is parvocellular biased, we predict no increase in suppression duration across contrast for 10 Hz maskers as they elicit magnocellular responses which saturate by 30% contrast. By contrast, suppression duration should rise gradually for 2 Hz maskers as parvocellular responses rise monotonically with contrast. A magnocellular bias will show no contrast effect, with 10 Hz consistently a more effective masker than 2 Hz. If CFS suppression shows neither bias, increasing contrast should increase masker power and monotonically increase suppression durations for both masker rates, with low more effective than high frequency. (**b**) Suppression duration as a function of masker contrast and temporal frequency. Consistent with a parvocellular bias, increasing masker contrast increased suppression duration, but only for the 2 Hz maskers. Paired-samples t-tests show significantly greater suppression for 2 Hz relative to 10 Hz maskers across all contrast levels. Asterisks denote statistical significance (**p < 0.01, ***p < 0.001) after Holm-Bonferroni correction. Error bars represent ±1 standard errors of the mean.

Masking stimuli were spatiotemporally narrowband, created by filtering 205 randomly generated noise images (each 128 × 128 pixels, approximately 5.4° by 5.4°). The stack of noise images was first converted to the frequency domain using a three-dimensional Fast Fourier Transform (FFT), before applying spatial and temporal filters. Spatially, each noise image was given a 1/f amplitude spectrum since our targets were natural images that have a 1/f amplitude spectrum^57^ (see also spatial spectrum of target images in Fig. 2a). In the temporal dimension, a log-Gaussian filter with a full bandwidth of one octave was used to sample temporal frequency into narrow passbands. The peak frequency was varied to sample temporal passbands of 0.375, 0.75, 1.5, 3, 6.25, 12.5 and 25 Hz. The filtered three-dimensional spectrum was then inversed transformed, resulting in a temporally narrowband, continuously varying pink noise stimulus as opposed to the discrete presentation of the Mondrian. As a control, static maskers were also used (a single noise image with 1/f spatial spectrum, randomly sampled every trial from the stack of 205 noise images). All noise images were normalised to maximum contrast (15% RMS) and a mean luminance spatial average. As an additional validation control, a standard 10 Hz Mondrian masker^38^ was used. Each pattern contained 265 squares of 0, 30, 50, 70 or 100% luminance that also varied in size from 0.52° to 1.30° in length. The Mondrian masker was presented in a similar fashion as the noise maskers and had the same dimensions (i.e., 5.4° by 5.4°), with the exception that the patterns were updated every 100 ms. All maskers were presented at 95% of maximum contrast.

### Target stimuli

For compatibility with previous studies, we used static natural images as targets (a total of four, including two images of man-made objects). Image categories were chosen to be as inclusive as possible, only excluding categories that are reportedly preferentially processed (e.g., faces)^58^,^59^. Without deeper understanding of preferential processing in CFS, this approach allowed us to keep the categories simple and task difficulty at a reasonable level. Image boundaries were smoothed with a two-dimensional Gaussian kernel with a standard deviation of 1.5 pixels. Targets were 2° by 2° in size and were presented at 30% of maximum contrast in one of four quadrants within a 5.4° by 5.4° area to the suppressed area. Target location within each quadrant was jittered with 1° steps from trial to trial to reduce predictability and local adaptation.

To ensure stable fusion of the left- and right-eye images, dichoptic targets and maskers were each surrounded by a checkerboard frame 0.5° thick measuring 5.9° by 5.9° externally and 5.4° by 5.4° internally. To avoid abrupt transients, the contrast of the maskers and targets gradually ramped up to the specified magnitude during the initial 1000 ms of each trial (Fig. 2c). The masker was presented 50 ms before the target to allow for the accumulation of suppressive effects from successive flashes^6^. All visual stimuli were presented via a DATAPixx video processor on a Mitsubishi Diamond Pro 2070SB CRT monitor with linearised luminance output at a screen refresh rate of 100 Hz and with 16-bit contrast resolution.

### Procedure

Participants viewed the dichoptic stimuli through a mirror stereoscope, which was individually adjusted to achieve stable fusion. During a trial, participants fixated a central cross while the target and masker ramped up over 1 s to their specified contrast and waited until the target emerged. Their task was to indicate, as accurate as possible, the quadrant where the target was located. After each trial, the time required for the target to reach visibility (suppression duration) was recorded, followed by 5 seconds of binocularly presented white noise to mitigate afterimages and adaptation effects. Each participant completed an average of 20 trials (10 trials per eye) for each masker temporal frequency, the order of which was randomised across participants. Presentation of target and masker was also randomised across dominant and non-dominant eyes to mitigate adaptation effects. To familiarise participants with the task demands, targets were presented at 60% of maximum contrast before the experimental task. For each masker frequency, suppression durations were computed by averaging the suppression durations for which the target quadrant was correctly identified. Any outliers larger than three times the median absolute deviation from the median were excluded. Each participant’s data were then normalised to their respective average durations across all masker temporal frequencies. The grand average per temporal frequency was then computed across all participants.

## Experiment 2

### Stimuli

Contrast detection and increment thresholds over temporal frequency were measured using the same masker patterns used in Experiment 1 except they were spatially windowed by a two-dimensional Gaussian function (*SD*_*xy*_ = 10 pixels), reducing the patterns to approximately 2° by 2° in size. These were monocularly presented (counterbalanced across eyes) 1.3° to the left or right of a central fixation cross within the same checkerboard frame as in Experiment 1. Targets were presented for 1 second, followed by 300 ms of visual white noise.

### Procedure

Participants first completed the detection threshold task followed by incremental thresholds measured with the standard stimuli presented at 2.5 times the absolute threshold. Their task was to indicate target location (left or right of fixation) in the detection task or the target with the higher contrast in the discrimination task. Each participant completed 32 trials per masker temporal frequency (16 per eye) with the order counterbalanced across participants. An adaptive staircase was used to vary contrast (QUEST) and thresholds were defined as the contrast at which responses were 75% accurate. To obtain a detection sensitivity curve, each participant’s thresholds were normalised to the respective average and then converted to the reciprocal value. Sensitivity curves for the increment task were computed from the just noticeable difference (JND) for each temporal frequency which were normalised to each participant’s average and converted to the reciprocal.

## Experiment 3

### Stimuli

As for Experiment 1 except that target stimuli were filtered into low spatial frequency (1 cpd) and high spatial frequency (10 cpd) components by convolving the target images with a log-Gaussian spatial filter with a 1-octave bandwidth.

### Procedure

As for Experiment 1 except that participants completed 16 trials per masker temporal frequency (8 trials per eye) in each spatial frequency condition, indicating the quadrant containing the target as soon as it became visible.

## Experiment 4

### Stimuli

Maskers were generated as in the previous experiments but only 2 levels of temporal frequency were compared: 2 and 10 Hz. Target stimuli were as in Experiment 1, presented at 25% of the maximum contrast while three levels of masker contrast were compared: 30, 50 and 90% of the maximum.

### Procedure

Sixteen trials per masker temporal frequency (8 trials per eye) were presented, the frequency order and eye of presentation were counterbalanced across participants. Task and data analysis as in Experiments 1 and 3.

## Additional Information

**How to cite this article**: Han, S. *et al*. The temporal frequency tuning of continuous flash suppression reveals peak suppression at very low frequencies. *Sci. Rep.* **6**, 35723; doi: 10.1038/srep35723 (2016).

## Figures and Tables

**Table 1:** Holm-Bonferroni corrected paired-sample tests in Experiment 3.

Masker frequency (Hz)	1 cpd	10 cpd	t-statistic	*df*	*p* value
0.375	0.66 (*SD* = 0.28)	1.65 (*SD* = 0.76)	3.63	11	< 0.05
0.75	0.78 (*SD* = 0.30)	2.27 (*SD* = 0.80)	5.87	11	< 0.0001
1.5	0.70 (*SD* = 0.26)	2.29 (*SD* = 1.38)	3.61	11	< 0.01
3	0.63 (*SD* = 0.17)	1.51 (*SD* = 0.65)	4.15	11	< 0.05
6.25	0.54 (*SD* = 0.26)	0.84 (*SD *=* *0.30)	4.42	11	< 0.01
12.5	0.47 (*SD* = 0.20)	0.65 (*SD* = 0.26)	3.87	11	< 0.05
25	0.47 (*SD* = 0.18)	0.55 (*SD *=* *0.18)	3.20	11	< 0.01

**Table 2:** Parameter estimates for Experiment 3.

Parameter	1 cpd	10 cpd	t-statistic	*df*	*p* value
Amplitude	0.38 (*SD* = 0.19)	2.38 (*SD* = 1.35)	4.46	9	< 0.01
Peak frequency (Hz)	0.99 (*SD* = 0.50)	0.95 (*SD* = 0.42)	0.99	9	0.86
Width (octaves)	1.33 (*SD* = 0.90)	1.53 (*SD* = 0.54)	0.86	9	1.0
Baseline	0.43 (*SD* = 0.20)	0.50 (*SD* = 0.25)	2.05	9	0.67
R^2^	0.85 (*SD* = 0.18)	0.92 (*SD *=* *0.09)	1.21	9	0.40
